# Rigid bioplastics shape the microbial communities involved in the treatment of the organic fraction of municipal solid waste

**DOI:** 10.3389/fmicb.2022.1035561

**Published:** 2022-11-11

**Authors:** Francesca Bandini, Filippo Vaccari, Mariangela Soldano, Sergio Piccinini, Chiara Misci, Gabriele Bellotti, Eren Taskin, Pier Sandro Cocconcelli, Edoardo Puglisi

**Affiliations:** ^1^Department for Sustainable Food Process, Università Cattolica del Sacro Cuore, Piacenza, PC, Italy; ^2^Centro Ricerche Produzioni Animali S.p.A. (CRPA), Reggio Emilia, RE, Italy

**Keywords:** biofilm, microbial ecology, anaerobic digestion, composting, metagenomics

## Abstract

While bioplastics are gaining wide interest in replacing conventional plastics, it is necessary to understand whether the treatment of the organic fraction of municipal solid waste (OFMSW) as an end-of-life option is compatible with their biodegradation and their possible role in shaping the microbial communities involved in the processes. In the present work, we assessed the microbiological impact of rigid polylactic acid (PLA) and starch-based bioplastics (SBB) spoons on the thermophilic anaerobic digestion and the aerobic composting of OFMSW under real plant conditions. In order to thoroughly evaluate the effect of PLA and SBB on the bacterial, archaeal, and fungal communities during the process, high-throughput sequencing (HTS) technology was carried out. The results suggest that bioplastics shape the communities’ structure, especially in the aerobic phase. Distinctive bacterial and fungal sequences were found for SBB compared to the positive control, which showed a more limited diversity. *Mucor racemosus* was especially abundant in composts from bioplastics’ treatment, whereas *Penicillium roqueforti* was found only in compost from PLA and *Thermomyces lanuginosus* in that from SBB. This work shed a light on the microbial communities involved in the OFMSW treatment with and without the presence of bioplastics, using a new approach to evaluate this end-of-life option.

## Introduction

Conventional petroleum-based plastics cover a wide range of applications (Koch and Mihalyi, 2018), and the annual European production in 2019 exceeded 57.9 million tonnes (Plastics Europe, 2020), leading to serious environmental pollution concerns due to the high amount of disposable items, reaching 29.1 million tonnes of post-consumer waste (Geyer et al., 2017; Rhodes, 2019). Consequently, micro- and nano-plastics, which are plastic particles derived from macro-plastics’ degradation with a diameter <5 mm and <100 nm, respectively (Karbalaei et al., 2018; Ng et al., 2018; Wang et al., 2019), are accumulating rapidly and have been already identified in different environments and places, such as Arctic ice, air, food and drinking water, soils, glaciers, and oceans, and also in human and animal bodies (Toussaint et al., 2019; Kanhai et al., 2020; Paul et al., 2020). While the possible impacts of micro- and nano-plastics on human health are still being investigated (Jiang et al., 2020), the increasing levels of plastic residues in the environment pose a new set of potential threats. To deal with these problems, bioplastics, including materials based on renewable sources, have arisen as possible beneficial alternatives (Abe et al., 2021) to reduce carbon footprint and dependency on fossil fuel, and to limit greenhouse gas emissions (Shamsuddin et al., 2017). Moreover, these polymers can be more easily biodegraded by a combination of abiotic processes, such as UV, temperature, moisture, and pH, as well as biotic parameters, mainly microbial activities (Mohee et al., 2008; Karamanlioglu et al., 2017; Harrison et al., 2018). According to European Bioplastics (2020), the current global bioplastics production slightly exceeds 0.6% of global plastic production, but the market is expected to grow in the upcoming years. Biodegradable plastics labeled as “compostable” must follow rigorous criteria as described in the European Standard EN 13432 (EN13432, 2002), in which, for instance, the breakdown under industrial composting conditions must occur in less than 12 weeks.

Among the different end-of-life options (i.e., recycling, landfill, etc.), the biological waste treatment, such as anaerobic digestion and the following aerobic composting of the organic fraction of municipal solid waste (OFMSW), is an environmentally safe and economically feasible treatment for bioplastics waste management (Butbunchu and Pathom-Aree, 2019). The industrial plants provide appropriate conditions for the efficient conversion of organic carbon into nutrient-rich compost that can be used as soil amender (i.e., compost). Moreover, energy recovery through anaerobic digestion is among the benefit of these worldwide developed processes (Song et al., 2009). However, the end-of-life options depend on the available waste management system in each country (Gómez and Michel Jr, 2013). In detail, anaerobic digestion leads to the production of biogas (mainly CH_4_ and CO_2_), water, hydrogen, sulfide, ammonia, and digestate through microbial metabolism and in absence of oxygen (Shrestha et al., 2020). The four microbiological reactions occurring, namely, hydrolysis, acidogenesis, acetogenesis, and methanogenesis, have been already extensively studied (Fernández-Rodríguez et al., 2013; Micolucci et al., 2016, 2018). On the one hand, the correct deterioration of organic matter, as well as bioplastics, may be influenced by process parameters, such as time and temperature. On the other hand, the aerobic composting process of the digestate consists of solid waste valorization (Kale et al., 2007; Spaccini et al., 2016) to obtain a homogeneous organic-rich and stable compost. This product, used as a soil amendment, has high microbial diversity (Song et al., 2009; Karamanlioglu and Robson, 2013; Sisto et al., 2018) and in composting plants the appropriate conditions for microorganisms growth are provided (Gu et al., 1993; Mohee et al., 2008; Sarasa et al., 2009; Gómez and Michel Jr, 2013; Javierre et al., 2015). The degradability of bioplastics is affected by different factors, such as their chemical and physical structures (Massardier-nageotte et al., 2006; Endres, 2017) and the high variety of biopolymer blends. The microbial diversity to which the bioplastics are exposed is among the most influential factors in degradation together with the operating conditions (Folino et al., 2020). For instance, according to the literature, the rates of degradation differ under aerobic and anaerobic conditions (Thakur et al., 2018), highlighting the relevance of microbial activity. Since the existing plants processing the OFMSW were not designed to also treat bioplastics, they may not be totally effective in the management and deterioration of these materials (Calabrò and Grosso, 2019). Furthermore, bioplastics may also play a key role in shaping the microbial communities involved during the processes (Bandini et al., 2020).

We reproduced at pilot-scale the anaerobic digestion and the following aerobic composting of the OFMSW treatment under real industrial conditions for time and temperature (25 days HRT at 52 ± 0.5°C and 22 days at 65 ± 0.2°C, respectively) (Bandini et al., 2022). Polylactic acid (PLA) and starch-based bioplastic (SBB) in the final form of rigid spoons, which are supposed to follow the same fate as the OFMSW, were tested at 5% (w/w) concentration on organic waste, separately, according to the latest report provided by Consorzio Italiano Compostatori (CIC-COREPLA, 2020). To monitor the bacterial, archaeal, and fungal communities during the whole process, high-throughput sequencing (HTS) technology of phylogenetic markers was carried out on the digestate used as inoculum, on the OFMSW slurry, on intermediate samples during the anaerobic digestion and on the final composts from each treatment. This study aims (i) to determine the possible influence of biopolymers on the microbial structure during the OFMSW treatment; (ii) to assess in detail the microbial evolution during anaerobic digestion, comparing the positive control (OFMSW only) with the two bioplastics; and (iii) to evaluate the microbiological quality of the final composts obtained from both the tested materials separately.

## Materials and methods

### Pilot-scale experiment setup and tested materials

The tested materials were compostable and commercially available PLA and SBB spoons with the same weight and thickness, which were broken into 2–5 mm pieces. The OFMSW slurry was sampled from a full-scale anaerobic and thermophilic plant. This slurry, which was previously subjected to separation from bioplastics and squeezing of the fraction, was further sifted with a 5-mm sieve to remove coarse fractions and plastics and to improve its homogeneity. The inoculum was collected from an OFMSW thermophilic plant and used immediately. All the substrates, including bioplastics, were chemically characterized as reported in a previous study (Bandini et al., 2022).

The anaerobic digestion was performed in three 24 L continuous stirred tank reactors under thermophilic conditions (52 ± 0.5°C). Each digester was filled with (i) OFMSW (positive control or blank) slurry and inoculum, (ii) OFMSW slurry, inoculum, and ground PLA spoons (2–5 mm), and (iii) OFMSW slurry, inoculum, and ground SBB spoons (2–5 mm). To reproduce the current Italian situation, the percentage of bioplastics to OFMSW was set at 5% w/w, and the reactors were daily fed. The hydraulic retention time (HRT) was 25 days, and the organic loading rate (OLR) was set based on chemical analyses. The following aerobic composting of the digestates lasted 22 days at 65 ± 0.2°C. The digestates from the three reactors were recovered by centrifugation and mixed with a green fraction of organic waste in a 1:1 ratio. Three technical replicates were obtained from each digestate for composting. A brief description of the experiment is given as an outline in Supplementary Figure 1.

### Anaerobic digestion and composting monitoring analyses

On the one hand, during anaerobic digestion, the quantity and quality of the produced biogas were monitored. The percentage in the volume of CH_4_, CO_2_, O_2_, and the concentration of H_2_S and organic acids were measured, and the temperature inside the reactors was continuously recorded. On the other hand, after the aerobic composting of the solid digestates, pH, humidity, and inert materials were determined together with the disintegration degree of the tested materials. The measurement of the bioplastic content in the final compost was performed in a fraction between 2 and 10 mm. The quality of the final composts was evaluated through phytotoxicity and ecotoxicity tests on seeds and soil fauna, respectively. Other physicochemical analyses on materials were performed and reported elsewhere (Bandini et al., 2022).

### DNA extraction, amplification, and sequencing

The DNA was extracted from the thermophilic anaerobic inoculum, the sieved OFMSW slurry, and the mix of inoculum and slurry. To monitor the microbial activity, intermediate sampling during the anaerobic digestion was carried out, namely at time 0 (T0), approximately one (T1) and two (T2) weeks after the addition of bioplastics to the reactors, and on the last day (Tfinal). The DNA was also extracted from the final composts obtained from the three treatments. The PowerSoil DNA Isolation Kit (Qiagen) was used, according to the manufacturer’s instructions. DNA quantification was performed with the Quant-iT HS ds-DNA assay kit (Invitrogen, Paisley, UK) using a QuBitTM fluorometer and stored at −20°C for further analyses. Analyses were carried out using three replicates for each sample.

### DNA amplification and Illumina high-throughput sequencing

To assess the bacterial, archaeal, and fungal communities, microbiological analyses based on HTS of 16S rDNA and Internal Transcribed Spacer 1 (ITS1) region of ribosomal RNA (rRNA) amplicons were performed, respectively.

The V3–V4 bacterial regions were amplified by PCR using universal primers 343F (5′-TACGGRAGGCAGCAG-3′) and 802R (5′-TACNVGGGTWTCTAATCC-3′). The PCR amplifications were performed with the Phusion Flash High-Fidelity Master Mix (Thermo Fisher Scientific, Inc., Waltham, MA, USA), and the mixture comprised 12.5 μl of Phusion Flash High-Fidelity Master Mix, 1.25 μl of each primer (10 μM), and 1 ng of DNA template and nuclease-free water, to a final volume of 25 μl. The thermocycler program was used as follows: initial denaturation at 95°C for 5 min, followed by 20 cycles of denaturation at 95°C for 30 s, annealing at 50°C for 30 s, an extension at 72°C for 30 s, and a final extension at 72°C for 10 min.

Differently, to assess the archaeal diversity, the V3–V4 regions were amplified using the primer pair 344F (5′-ACGGGGYGCAGCAGGCGCGA-3′) (Raskin et al., 1994) and 806R (5′-GGACTACVSGGGTATCTAAT-3′) (Takai and Horikoshi, 2000). The PCR reaction mix was composed of 12.5 μl of Phusion Flash High-Fidelity Master Mix, 1.25 μl of each primer (10 μM), 1 ng of DNA template, and nuclease-free water, to a final volume of 25 μl. The thermocycler program was with the following conditions: initial denaturation at 94°C for 5 min; followed by 20 cycles of denaturation at 90°C for 30 s, annealing at 50°C for 30 s, an extension at 72°C for 30 s, and a final extension at 72°C for 10 min.

Finally, the fungal communities were estimated using the universal primers ITS-1 (5′-TCCGTAGGTGAACCTGCGG-3′) and ITS-2 (5′-GCTGCGTTCTTCATCGATGC-3′; White et al., 1990), and PCR reactions were carried out with 12.5 μl of Phusion Flash High-Fidelity Master Mix, 1.25 μl of each primer (10 μM), and 1 ng of DNA template and nuclease-free water, to a final volume of 25 μl. The thermocycler was set with an initial hold at 94°C for 4 min, followed by 28 cycles of 94°C for 30 s, annealing at 56°C for 30 s, an extension at 72°C for 1 min, and a final extension at 72°C for 7 min.

To allow simultaneous analyses and reduce the generation of anomalous data, an indexing PCR was performed. Amplicons were combined in equimolar ratios and multiplexed into two separate pools, one for Bacteria and Archaea and another for Fungi. The pools were purified throughout the solid-phase reversible immobilization (SPRI) method (Agencourt AMPure XP kit; REF A63880, Beckman Coulter, Milano, Italy) and sequenced by Fasteris S.A. (Geneva, Switzerland). The amplicon libraries were prepared using the TruSeq DNA sample preparation kit (REF 15 026 486, Illumina Inc., San Diego, CA, USA), and the sequencing was carried out with the MiSeq Illumina instrument (Illumina Inc.), generating 300 bp paired-end reads.

### Sequence processing and statistical analyses

Data filtering, multiplexing, and preparation for statistical analyses were performed according to previous studies (Berry et al., 2011; Vasileiadis et al., 2015; Bortolini et al., 2016; Puglisi et al., 2019; Bandini et al., 2020). Briefly, the MiSeq Control Software version 2.3.0.3, RTA v1.18.42.0 and CASAVA v1.8.2 were used for base calling, whereas the “pandaseq” script was applied to align raw Illumina sequences (Bartram et al., 2011). Since V3-V4, 16S rRNA and ITS1 gene amplicons are shorter than 500 bp and require 300 bp paired-end reads per amplicon for re-constructing full regions, a minimum overlap of 30 bp between read pairs and two maximum allowed mismatches was set. Fastx-toolkit^1^) was applied to demultiplex the sequences according to sample indexes and primers. According to Bortolini et al. (2016), among the bacterial and archaeal sequences, homopolymers >10 bp (chimeras), sequences outside the regions of interest, and non-targeted taxa were removed. The UCHIME algorithm with the UNITE database v6 was used to identify and discard homopolymers >10 bp, chimeras, and non-fungal sequences among the ITS amplicons. Operational taxonomic units (OTUs) and taxonomy-based approaches were performed on the sequences. Mothur V1.32.1 was applied for the OTUs and taxonomy matrixes for the V3–V4 regions (Schloss et al., 2009), whereas statistical analyses were performed with R^2^ supplemented with Vegan package (Dixon, 2003). OTUs were determined in VSEARCH. Mothur was used to perform ITS taxonomy-based analyses with a minimum length of 120 bp and no upper length limit due to ITS variability. The average linkage algorithm was applied at different taxonomic levels to visualize the hierarchical clustering of the sequences, while the unconstrained sample grouping was assessed by principal component analysis (PCA), and the canonical correspondence analysis (CCA) was used to visualize the significance of different treatments on the analyzed diversity (constrained variance).

To estimate the associated α and β diversity, the OTU- and taxonomy-based matrixes were analyzed using R. Moreover, to better estimate any significant differences between samples, α diversity analyses based on Shannon’s Index, Observed Richness (S), Simpson’s Diversity Index (D), and Chao’s Index were performed on bacterial and fungal communities as previously described (Bandini et al., 2020). Good’s coverage estimate was calculated to assess the “percentage diversity” captured by sequencing (Good, 1953), whereas to identify significantly different features between samples Metastats coupled to FDR test for the comparison of means was applied (Paulson et al., 2011). The identification of the most abundant OTUs was confirmed with RDP (Ribosomal Database Project) for Bacteria and Archaea and with BLAST (Basic Local Alignment Search) searches of the GenBank database for Fungi. Analysis of variance of means (ANOVA) and Tukey’s HSD pairwise comparison test (α < 0.05) were performed to assess significant differences between samples. The raw sequences data have been deposited in the Sequence Read Archive of NCBI, and the accession numbers will be assigned.

In the experiment and data analyses, samples were labeled as follows: digestate used as inoculum, “T0_none_initial_ digestate”; OFMSW slurry, “T0_none_OFMSW_slurry”; the mixture of digestate and OFMSW slurry as initial time T0, “T0_none_digestate_OFMSW”; intermediate samples at T1, T2, and Tfinal during anaerobic digestion for each treatment, “TX_treatment_anaerobic”; final compost from each treatment, “Tfinal_treatment_compost”. Each sample was reported in triplicate.

## Results and discussion

### Thermophilic anaerobic digestion and aerobic composting of rigid bioplastics with organic fraction of municipal solid waste

Probably, due to the high daily OLR, the specific methane production for bioplastics was lower than the positive control, and biological process problems were found after 2 weeks (Bandini et al., 2022). The process instability also resulted in high H_2_S concentration in the gas phase in the last period of the test and in a progressive increase over time in the concentration of organic acids. At the end of the anaerobic digestion with OFMSW, PLA and SBB spoon residues remained in an undegraded form leading to serious risks to clog pipers or the mechanical parts of the reactors. Also, after the aerobic composting, the tested bioplastics did not reach the disintegration target imposed by UNI EN 13432 standard, and several residues were found in the final compost. Phytotoxicity tests reported the lowest Germination Index for PLA elutriate, whereas a potential negative effect of SBB on soil fauna was detected.

### Overview of the bacterial, archaeal, and fungal communities’ structure during organic fraction of municipal solid waste treatment with bioplastics

**FIGURE 1:**
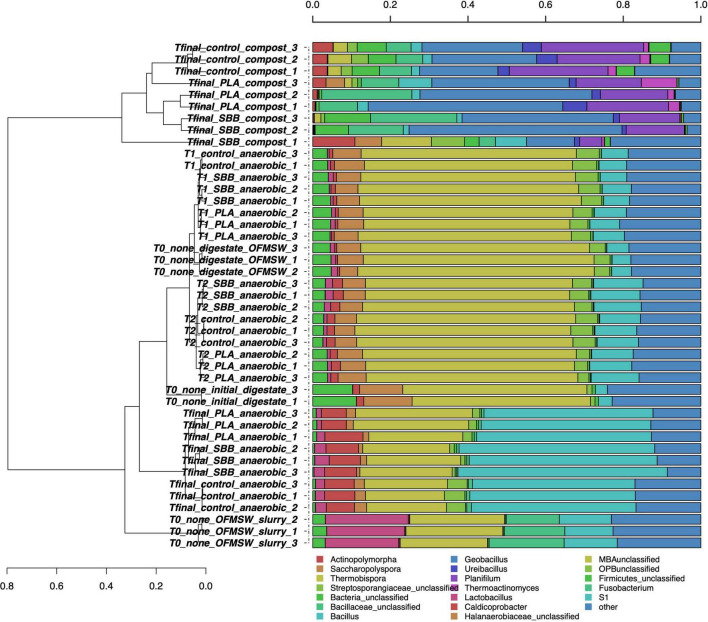
Hierarchical clustering of bacterial sequences using the average linkage algorithm at the genus level for taxa with at least ≥5% participation in a single sample. Taxa with low contributions were added to the “other” group. PLA, polylactic acid; SBB, starch-based bioplastic; OFMSW, organic fraction of municipal solid waste.

After the screening and filtering steps, bacterial amplicons resulted in 14,500 high-quality sequences per sample and the dataset revealed a consistent coverage of 99%. Sequences were classified at 51.6% to family level, at 43.1% to genus level, and at 5.9% to species level (data not shown). Figure 1 shows the hierarchical clustering of classified sequences using the average linkage algorithm at the genus classification level for all the samples. The samples clustered according to treatments, showing differences in bacterial community between the OFMSW slurry and all the other matrixes. The digestate was located between the intermediate samples of T2 and the Tfinal of anaerobic digestion of the PLA treatment, exhibiting nevertheless a unique diversity. Probably due to the microbial richness of the digestate, the initial mixture T0 clustered among the T1 of PLA and the T2 of SBB. The composts were placed together and showed a wider bacterial diversity than the anaerobic digestion samples. Even among the same treatment, some differences further demonstrate the microbial diversity of these samples. However, the final compost from OFMSW showed a different bacterial diversity compared to the two tested bioplastics. The most abundant genera in OFMSW slurry were *Lactobacillus* and *Fusobacterium*, and these were not found in any other treatments. OTUs assigned to the Halanaerobiaceae family were found mainly in the initial digestate and in almost all anaerobic digestion samples, although the abundance decreased from T0 to T2 until it dropped significantly at Tfinal for the three treatments without differences between the positive controls and the bioplastics. This family contains fermenting and cellulolytic bacteria (Simankova et al., 1993) and was already reported in the thermophilic digester (Li et al., 2015). *Caldicoprobacter* was the distinctive genus of the anaerobic digestion Tfinal samples where it was found with higher abundance and without significant differences between OFMSW and bioplastics treatment. Bacteria of this thermophilic genus belonging to the class *Clostridia* are known for fermenting sugars into acetate, lactate, ethanol, hydrogen, and carbon dioxide and were already detected in anaerobic digestion (Ziganshina et al., 2017). Since they are obligate heterotrophic bacteria mainly isolated from hydrothermal hot springs (Bouanane-Darenfed et al., 2013), and their presence at the end of the anaerobic phase may be due to their resistance to high ammonia levels. *Bacillus, Geobacillus*, and *Ureibacillus* were the dominant genera in all compost samples and were already reported in literature for mature substrate after composting (Vajna et al., 2012). These thermophilic bacteria are frequently detected in compost as they contribute to the vigorous degradation of organic compounds (Hanajima et al., 2009), such as *Ureibacillus*, which is capable of degrading lignocellulose (Wang et al., 2011) and was also isolated during thermophilic composting of sludge (Steger et al., 2005). Their mutual presence in the compost from OFMSW, PLA, and SBB may suggest that these microorganisms contribute significantly to the degradation of cellulosic material, and possibly bioplastics, during the thermophilic composting phase of the anaerobic digestates. Differently, *Actinopolymorpha* and *Thermobispora* genera were mainly found in compost obtained from OFMSW, and the second one was already isolated from this substrate (Steger et al., 2007; Cao et al., 2019). *Thermoactinomyces* genus was detected especially in compost obtained from OFMSW and PLA. Since a member of this thermophilic and lipolytic actinomycete was inoculated in compost to more efficiently decompose food waste into mature substrate (Ke et al., 2010), its presence in composts from PLA may be positively evaluated.

**FIGURE 2:**
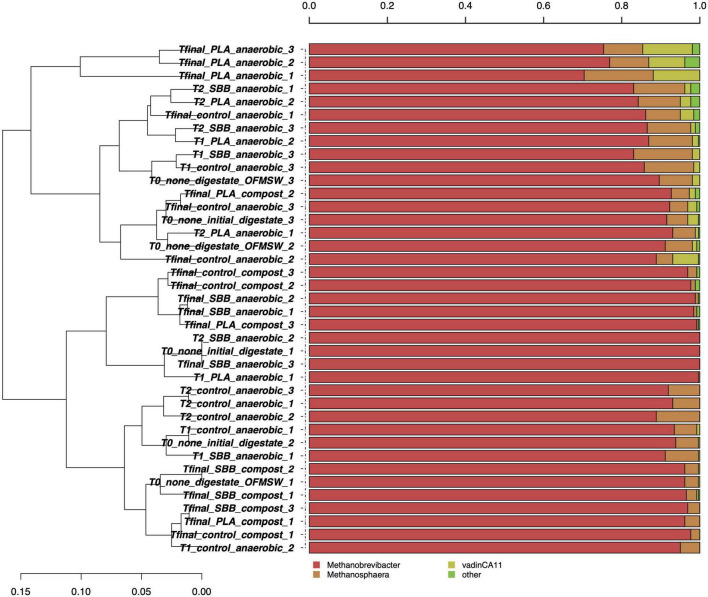
Hierarchical clustering of archaeal sequences using the average linkage algorithm at the genus level for taxa with at least ≥5% participation in a single sample. Taxa with low contributions were added to the “other” group. PLA, polylactic acid; SBB, starch-based bioplastic; OFMSW, organic fraction of municipal solid waste.

**FIGURE 3:**
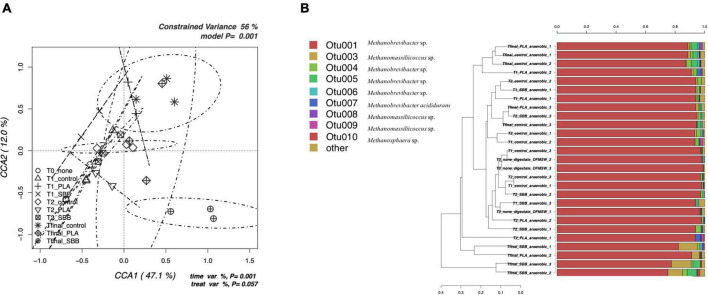
Archaeal community during anaerobic digestion. **(A)** Hypothesis-driven canonical correspondence analysis (CCA) model performed on the total archaeal OTUs relative abundance data from anaerobic digestion intermediate samples. **(B)** Overview and identification of the most abundant OTUs comprising 99% of the archaeal diversity in anaerobic digestion intermediate samples. PLA, polylactic acid; SBB, starch-based bioplastic; OFMSW, organic fraction of municipal solid waste.

Figure 2 shows the taxonomic assignment at the genus level for the 261 archaeal high-quality sequences per sample. The average coverage rate was 98%, and the sequences were classified into family (100%), genus (99.8%), and species (1.2%) levels (data not shown). Such a low taxonomic assignment at the species level could be explained by the scarcity of data deposited in the main database. As will later be shown in Figure 3, one OTU was identified at the species level. Since these microorganisms are hardly cultivable under laboratory conditions and are often associated with extreme growth conditions, their knowledge is still limited. In Figure 2, the most abundant archaeal genera are shown. *Methanobrevibacter* and *Methanosphaera* were the only genera identified, and no particular differences were observed between the samples in the overall reproduced OFMSW process. *Methanobrevibacter* was the dominant genus in each sample and was positively correlated with biogas yield playing a key role in biogas production (Li et al., 2015). This genus belonging to the Methanobacteriaceae family generally dominates the biogas reactors and was also present in fresh cattle manure (Goberna et al., 2015). High concentration of H_2_S detected in the reactors may have significantly modulated the archaea biodiversity during anaerobic digestion (Bandini et al., 2022). As reported in a recently published research, since H_2_S is toxic to methanogens, there is a strong correlation between the presence of this compound and the microbial population in the reactors (Olivera et al., 2022). Otherwise, the *Methanosphaera* genus was detected in each sample except some as compost from PLA, T2 for SBB treatment, one replicate of digestate, and Tfinal for SBB. The presence of this methanogenic archaea was already reported in literature (Goberna et al., 2015; Achmon et al., 2019) and dominated the active methane production during co-digestion of food waste (Zhang et al., 2016). Members of this genus have one of the most restricted metabolisms since they can neither oxidize methanol to carbon dioxide nor reduce carbon dioxide to methane (Fricke et al., 2006), and methanol- and acetate-rich environments provide the optimum conditions to grow (Facey et al., 2012). However, we cannot exclude the hypothesis that the presence of archaeal DNA in composting samples can be a residue from the anaerobic phase.

**FIGURE 4:**
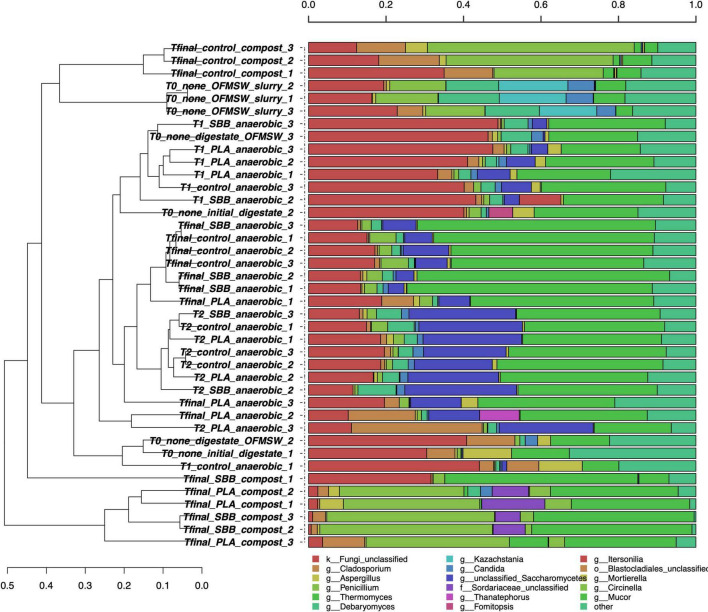
Hierarchical clustering of fungal sequences using the average linkage algorithm at the genus level for taxa with at least ≥5% participation in a single sample. Taxa with low contributions were added to the “other” group. PLA, polylactic acid; SBB, starch-based bioplastic; OFMSW, organic fraction of municipal solid waste.

Figure 4 shows the taxonomic classification of the 12,500 ITS1 sequences per sample. The Good’s coverage result was 99%, and the sequences were classified at 75.9% at the family level, 74.5% at the genus level, and 65.7% at the species level. The OFMSW slurry presented a separate fungal community, dominated by *Penicillium*, *Debaryomyces*, *Kazachstania*, and *Candida* genera. *Penicillium* species usually govern food waste (Torp and Skaar, 1998; Rundberget et al., 2004), whereas *Kazachstania* spp. was already found in food waste slurry (Wang et al., 2020) and traditional fermentation, such as alcohols production, is connected to this genus (Di Cagno et al., 2014; Lhomme et al., 2015). Otherwise, the anaerobic digestion samples, including the initial digestate and intermediate times of all the treatments, did not cluster uniformly between replicates and stages. However, some dominant fungi can be identified only in these samples, such as unclassified *Saccharomycetes*, *Cladosporium*, and *Mortierella*. Due to the high levels of proteins, amino acids, and ammonia food waste is an adequate substrate for the growth of *Saccharomycetes* (Suwannarat and Ritchie, 2015), which was also applied for the anaerobic digestion of kitchen waste (Zheng et al., 2022). Differently, compost samples were grouped into separate clusters, showing a heterogeneous trend. The positive control was distantly related to the two bioplastic-treated composts, exhibiting a completely different fungal community. Fungi are mainly active in an aerobic environment and they also play a crucial role in the polymers’ biodegradation process (Folino et al., 2020). In the compost from OFMSW, *Cladosporium*, *Penicillium*, and *Mucor* were the dominant fungal genera. In recent work, *Cladosporium* was found during composting of digestate from food waste and it is associated with the ability to degrade lignocellulose matter (Ryckeboer et al., 2003). The presence of this fungus in compost from OFMSW could be a good indicator. It was also found in compost treated with PLA and SBB, but in lower abundance than the positive control. The unclassified *Sordariaceae* genus was instead a characteristic genus in compost from SBB and PLA, and this membranous or coriaceous ascomata fungus was often found on decaying substrate, such as wood (Zhang et al., 2006; Maharachchikumbura et al., 2016). In conclusion, especially in the final composts, strong differences can be noticed between the positive control and the bioplastics treatments, highlighting a possible effect of these materials in modulating the fungal community involved in the aerobic process.

Based on the HTS results, detailed analyses were carried out in terms of α-diversity, namely, the distribution of the most abundant bacterial, archaeal, and fungal OTUs. Chao, Shannon’s, Simpson’s (D), and Observed Richness (Sobs) α-diversity indexes were calculated to evaluate the diversity, the evenness, and the dissimilarity in terms of community structure between the microbial communities at different stages and with different treatments. Supplementary Table 1 reports the average (±SD) of α-diversity indexes and richness of bacterial microbiomes. Based on ANOVA and LSD test (*p* < 0.05) performed, Chao, Sobs, and Shannon indexes on the bacterial α-diversity resulted in significant differences according to *F* values. In particular, the digestate and OFMSW slurry resulted in a different bacterial community compared to all other samples (Chao and Sobs). The intermediate anaerobic digestion samples showed fewer differences between stages and treatments. Otherwise, archaeal α-diversity indexes (Supplementary Table 2) resulted in significant differences for Sobs, Simpson, and Shannon. The digestate reported a significantly different archaeal community structure, whereas lower distinctions were appreciated for the other samples. According to Sobs, Shannon, and Chao indexes, the OFMSW and PLA composts were significantly lower compared to SBB. As for bacteria, the fungal Chao, Sobs, and Shannon α-diversity indexes (Supplementary Table 3) resulted in significant differences. Composts from PLA and SBB reported significantly lower results for Chao, Sobs, and Shannon indexes compared to the compost from OFMSW. According to the Simpson’s Index, the T0 of anaerobic digestion reported significantly higher differences among other samples.

This overview of the bacterial, archaeal, and fungal communities involved in the digestate, in the OFMSW slurry, in different stages of anaerobic digestion, and in the final compost was crucial to assess their dynamics and the hypothetical role of bioplastics in modulating them. According to our knowledge, this is the first study to thoroughly map the microbial communities involved during the OFMSW treatment.

### A detailed focus on the bacterial community during thermophilic anaerobic digestion of organic fraction of municipal solid waste and bioplastic

**FIGURE 5:**
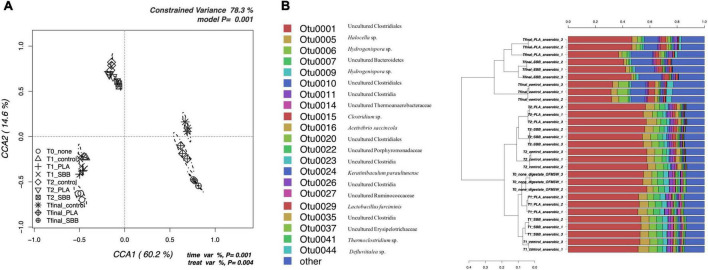
Bacterial community during anaerobic digestion. **(A)** Hypothesis-driven canonical correspondence analysis (CCA) model performed on the total bacterial OTUs relative abundance data from anaerobic digestion intermediate samples. **(B)** Overview and identification of the most abundant OTUs comprising 99% of the bacterial diversity in anaerobic digestion intermediate samples. PLA, polylactic acid; SBB, starch-based bioplastic; OFMSW, organic fraction of municipal solid waste.

To better evaluate the effects of bioplastics on the bacterial community during anaerobic digestion, a db-RDA model based on OTUs was applied. Figure 5A shows the CCA that was significant (*p* = 0.001) and had a high explanation of variance (78.3% of the total variance). The first and the second canonical axes represented 60.2 and 14.6% of the variance, respectively. T0 samples were negatively correlated with both axes and grouped separately, highlighting a distinct bacterial diversity. In the same sector, CCA were also located the samples of T1, showing a similar community between treatments and not very distant from that of T0. The T2 samples clustered together and overlapped the different treatments, whereas Tfinal showed different communities for positive control and bioplastics treatment.

Figure 5B represents the hierarchical cluster of the most abundant OTUs in anaerobic digestion samples and the respective identification confirmed with RDP. OTU0001, which corresponds to Uncultured Clostridiales, was predominant at all stages, but reduced toward the end of the anaerobic process, as reported in a recent study under thermophilic conditions (Jackson et al., 2020). In the early stages, OTU0001 was almost 60% of the total bacterial diversity as described in literature (Granada et al., 2018), reducing to 40–50% at Tfinal for SBB and PLA, and up to 30% in the positive control. In agreement with these results, it appears that both PLA and SBB have an effect on the abundance of Clostridiales, which are decisive performers during hydrolysis, acidogenesis, acetogenesis, and syntrophic acetate oxidation (Ziganshin et al., 2013). Both OTU0006 and OTU0009 were identified as *Hydrogenispora* sp., which are hydrolytic, acidogenic, and acetogenic bacteria already detected in anaerobic digester (Kang et al., 2021). These bacteria are able to ferment carbohydrates such as glucose, maltose, and fructose into acetate, ethanol, and hydrogen, respectively (Yang et al., 2016; Corbellini et al., 2018), and probably for this reason OTU0009 was slightly more abundant in T1 samples, especially in SBB. Otherwise, several strains of *Clostridium* sp. (OTU0015), mainly found in T2 and Tfinal of each sample, are key actors involved in the production of butyric acid as the main product of sugar metabolism (Soh et al., 1991). They also produce hydrogen, carbon dioxide, acetate, ethanol, and lactate (Kim et al., 2010) and, probably due to the ability of any strains to consume starch (Zhang et al., 2020), OTU0010, identified as Uncultured Clostridiales, was mainly found in the Tfinal of SBB treatment. OTU0016, identified as *Acetivibrio saccincola* (synonym: *Herbivorax saccincola*) was detected in all samples, although with a slightly lower abundance in Tfinal for PLA and SBB. This novel thermophilic and anaerobic bacterium was isolated from a lab-scale biogas fermenter and seems to play a key role in the remineralization of plant biomass by hydrolyzing polysaccharides (Koeck et al., 2016). This may also have important implications in the degradation of organic matter in bioplastics, and its lower abundance compared to the positive control may not be a good indicator. Finally, *Defluviitalea* sp. (OTU0044) was mainly found not only in OFMSW Tfinal but also in PLA. This spore-forming and the saccharolytic bacterium were already isolated from an anaerobic digester (Ma et al., 2017) and played a remarkable role in the digestion stage of acidogenesis (Zhang et al., 2017). Its absence in the Tfinal of the SBB treatment may not have a positive implication in the anaerobic digestion and degradation of organic matter processes.

### A detailed focus on the archaeal community during thermophilic anaerobic digestion of organic fraction of municipal solid waste and bioplastics

Figure 3A shows the CCA for the archaeal community structure during anaerobic digestion. The CCA model was significant (*p* = 0.001) and had a high explanation of variance (56% of the total variance). The first and the second canonical axes represented 67.1 and 12.0% of the variance, respectively. However, as reported in a previous study (Bandini et al., 2020), the archaeal population did not follow heterogeneous trend during the anaerobic digestion. Samples were overlapping for both different stages and treatments, with the only exception of the Tfinal for SBB, which showed more distinctive diversity. Indeed, the ellipse is completely confined in the sector positively correlated with the first axis and negatively correlated with the second axis and overlaps only with the archaeal diversity found in the Tfinal for PLA treatment.

The taxonomic assignment of the most abundant OTUs is reported in Figure 3B and, as mentioned in the overview discussed in the previous section, the diversity is limited. OTU001, identified as *Methanobrevibacter* sp., was dominant in each sample, especially in the digestate and in early fermentation samples. The abundance of this OTU was almost 100%, then dropping to about 70% for the SBB Tfinal. This genus is a methanogen responsible for methane production from hydrogen and carbon dioxide inside the process (Sousa et al., 2007; Wang et al., 2018) and was already detected in thermophilic conditions (Lee et al., 2014). The decrease in abundance in the final samples of the process may indicate a complete utilization of the substrates necessary for the growth of these archaea, such as biogas exhaustion. On the one hand, Other OTUs also belong to the *Methanobrevibacter* sp., but the most distinctive ones were OTU004 and OTU005 that were found mainly in the Tfinal of OFMSW and SBB. In general, PLA Tfinal did not show uniform diversity and clustered heterogeneously. On the other hand, Tfinal from PLA (a replicate) and SBB clustered differently compared to the positive control and reported a higher abundance of OTU003 (*Methanomassiliicoccus* sp.). This hydrogenotrophic genus was detected in the thermophilic anaerobic digester (Jackson et al., 2020; Sposob et al., 2020). Other OTUs that corresponded to the same genus were also found especially in the last stages of anaerobic digestion, without distinction between treatments, such as OTU008 which was detected in a replicate in the Tfinal of the positive control. As already reported in a previous study at lab-scale (Bandini et al., 2020) and in a recently published work (Peng et al., 2022), the relative abundance of the archaeal community is strictly related to the *Clostridium* genus. The growth of these hydrogenotrophic and syntrophic acetate-oxidizing (SAO) microorganisms increased in tandem with the *Clostridium* sp.

### A detailed focus on the fungal community during thermophilic anaerobic digestion of organic fraction of municipal solid waste and bioplastics

**FIGURE 6:**
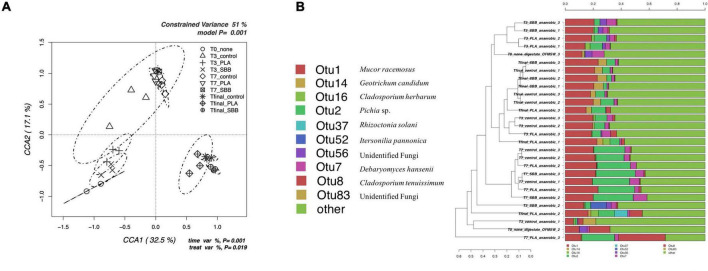
Fungal community during anaerobic digestion. **(A)** Hypothesis-driven canonical correspondence analysis (CCA) model performed on the total bacterial OTUs relative abundance data from anaerobic digestion intermediate samples. **(B)** Overview and identification of the most abundant OTUs comprising 95% of the fungal diversity in anaerobic digestion intermediate samples. PLA, polylactic acid; SBB, starch-based bioplastic; OFMSW, organic fraction of municipal solid waste.

The db-RDA model based on fungal OTUs reported in Figure 6A shows the CCA that was significant (*p* = 0.001) and had a high explanation of variance (51% of the total variance). The first and the second canonical axes represented 32.5 and 17.1% of the variance, respectively. The samples were quite heterogeneously distributed. In particular, the initial digestate was negatively correlated with both axes, almost completely overlapping with the T1 of PLA and SBB, probably showing a similar microbial community. The T1 of the positive control had a wide ellipsis, including both T1 samples from the two bioplastics and all T2 samples. The latter, in fact, were overlaid regardless of treatment, assuming a fairly similar fungal diversity. The final anaerobic digestion samples were positively correlated with the first axis and negatively correlated with the second axis, respectively. Those in the PLA treatment showed a broader community more similar to that of the positive control, whereas those in the SBB treatment reported a narrower and more distinct ellipse.

Figure 6B shows the hierarchical clustering of fungal sequences at the genus level. Studies of fungal populations involved during anaerobic digestion in the literature are scarce. However, given their affinity for acidic environments (Oranusi and Dahunsi, 2013), it is critical to understand whether they too play a key role in the fermentation of organic matter and, especially, whether they are affected by the presence of bioplastics during the process. Moreover, the biodegradation of bioplastics involves, together with prokaryotic, also eukaryotic fungi and protozoa microorganisms (Rujnić-Sokele and Pilipović, 2017). From Figure 6B, it can be noticed that not all replicates of the same sample are always grouped together, but in general, the clusters were in accordance with what is shown in Figure 6A. OTU1, identified as *Mucor racemosus*, was dominant in all samples but with lower abundance in the anaerobic digestate used as inoculum. Only in the T2 samples from all treatments, this OTU competed with the abundance of OTU2. This genus was already found previously in thermophilic anaerobic digesters treating food waste and human excreta (Oranusi and Dahunsi, 2013; Bandini et al., 2020). Moreover, this species was tested for phosphorous removal during waste stream (Ye et al., 2015) but was mainly isolated from mesophilic and thermophilic composting (Ryckeboer et al., 2003). *Geotrichum candidum* (OTU14) was detected only in Tfinal samples from all treatments, but in greater abundance in the reactor with SBB. This yeast is widely spread in the environment and has the ability to ferment carbohydrates, ethanol, and glycerol in anaerobic conditions (Fisgativa et al., 2017). *G. candidum* is also a plant pathogen, isolated from food waste (Fisgativa et al., 2017), which is particularly relevant for its strong lipase and protease activity on organic matter, and fatty acids and peptides production. Its higher abundance with the SBB treatment may be caused by the higher carbohydrate content of the SBB. OTU2, identified as *Pichia* sp. was detected in T1 and T2 indistinctly, although slightly with less abundance in SBB treatment. However, in general, the abundance of this genus increases greatly from the samples of the third day to those of the seventh day. *Pichia* sp. was found also in Tfinal samples in the same abundance as the T1 samples. This fermentative yeast had a facultative aerobic metabolism and was also found in a recent study focusing on fungal dynamics in the anaerobic digestion of sewage sludge and food waste (Qin et al., 2021). Moreover, this research states that fungal diversity varied in relation to HRT and OLR. *Pichia* sp. was particularly abundant after 34 days of anaerobic digestion, and this result agrees with our data which reported this genus mainly at T2 (after 18 days of reactor stabilization and 14 days after the addition of bioplastics, for a total of 32 days). Differently, *Rhizoctonia solani* (OTU37) and *Itersonilia pannonica* (OTU52) can be outlined among the most distinctive OTUs for one replicate of Tfinal from PLA and one replicate of T1 from SBB, respectively. *R. solani* is a common soil-borne fungal pathogen that was already isolated in feedstock (Bandte et al., 2013). According to this article, the anaerobic digestion at mesophilic conditions reduces most of the phytopathogens infecting plants, and it was remarkable to detect its presence at the end of the thermophilic treatment (Tfinal from PLA treatment). Finally, since one strain of *Cladosporium* sp. was identified as PLA-degrading microorganism (Nair et al., 2016), the abundance of OTU16 and OTU8 in PLA samples may play a key role in the degradation process of this biopolymer during the anaerobic digestion.

### Effects of polylactic acid and starch-based bioplastic on bacterial diversity in compost

**FIGURE 7:**
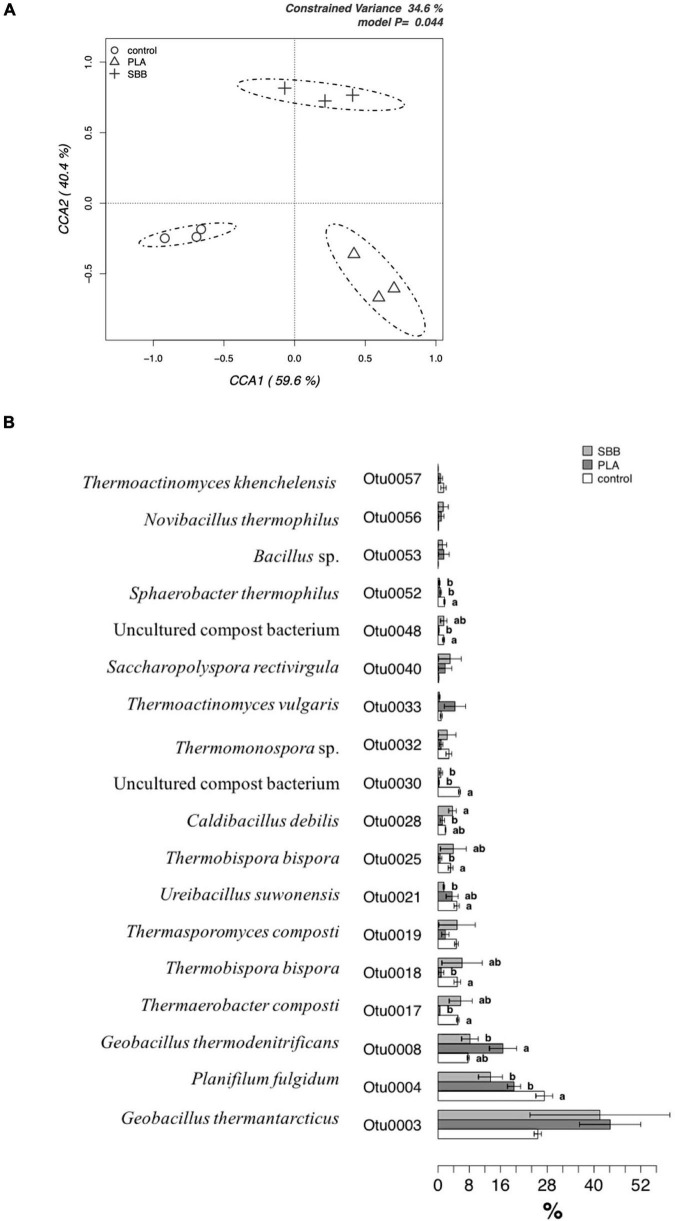
The bacterial community in compost. **(A)** Hypothesis-driven canonical correspondence analysis (CCA) model performed on the total bacterial OTUs relative abundance data from composts of each treatment (OFMSW, control; PLA; SBB). **(B)** Metastats model on the relative abundances of genera comprising 99.9% of each treatment’s bacterial diversity in composts (OFMSW, control; PLA; SBB). OTUs showing significant differences according to the false discovery rate correction are highlighted with letters. PLA, polylactic acid; SBB, starch-based bioplastic; OFMSW, organic fraction of municipal solid waste.

Composting is an aerobic biodegradative process of organic matter that involves numerous microorganisms. Microbial communities degrade the organic substrates into more stable, humified forms and inorganic products, while their succession is a prerequisite to ensure not only complete biodegradation (Ryckeboer et al., 2003) but also the quality of the final product. Figure 7A shows the CCA for the bacterial community structure in composts from the three treatments. The CCA model was significant (*p* = 0.044) and had a 34.6% of explanation of variance. The first and the second canonical axes represented 59.6 and 40.4% of the variance, respectively. As can be seen, the bacterial diversity differed significantly between samples obtained from OFMSW, OFMSW mixed with PLA, and OFMSW mixed with SBB. The positive control clustered negatively with both the axes, whereas the PLA and SBB were both positively correlated with the CCA1, but negatively and positively with the CCA2, respectively.

The Metastats model for the most abundant 15 OTUs is shown in Figure 7B. First of all, it is important to underline that after the composting phase no OTU previously identified in the anaerobic phase were found again. This is probably due to the difference between anaerobic and aerobic phases, both occurring at thermophilic conditions, which allowed the selection of different bacteria. Among the OTUs, *Geobacillus thermantarcticus* (OTU0003) was one of the most abundants, accounting for more than 40% of the sequences for composts obtained from both the bioplastics and about 28% for the positive control. To our knowledge, there are no references to this thermophilic strain in composts from the literature. However, its presence may be associated with its optimum growth at 60°C and its strictly aerobic metabolism (Coorevits et al., 2012). Statistically significant difference was found for OTU0004 and identified as *Planifilum fulgidum.* This microorganism, able to degrade hemicellulose, cellulose, and protein, was already detected as dominant in compost containing spent mushroom substrate and a high-nitrogen environment (Zhang et al., 2021). Other species belonging to the genus were found in compost, but its presence may be related to the presence of nitrogen. Indeed, the OTU0008 was also identified as *Geobacillus thermodenitrificans*, a thermophilic bacteria isolated from compost heap which have the ability to convert lignocellulose into lactic acid and reduce nitrate (Daas et al., 2018). This OTU was particularly abundant in the compost from PLA and significantly lower in that obtained from SBB, probably due to the difference in the availability of substrates, such as nitrogen. Moreover, a strain of *Geobacillus* is reported as an L-PLA degrading thermophile (Tomita et al., 2004). *Thermaerobacter composti* (OTU0017), already isolated from compost in Japan (Yabe et al., 2009), was more abundant in positive control and SBB compost compared to PLA, as well as OTU0018 (*Thermobispora bispora*), OTU0019 (*Thermasporomyces composti*), OTU0025 (*T. bispora*), and OTU0028 (*Caldibacillus debilis*). It is important to underline that no abundant sequences of pathogenic bacteria were found. Moreover, there were notably statistically significant differences between the compost samples obtained from the different treatments. In conclusion, the presence of bioplastics may greatly influence aerobic composting.

### Effects of polylactic acid and starch-based bioplastic on fungal diversity in compost

Some polymers are mainly, or even only, degraded by fungi (Rujnić-Sokele and Pilipović, 2017), and thus this microbial group will, therefore, play a key role during the aerobic composting of food waste and bioplastics.

**FIGURE 8:**
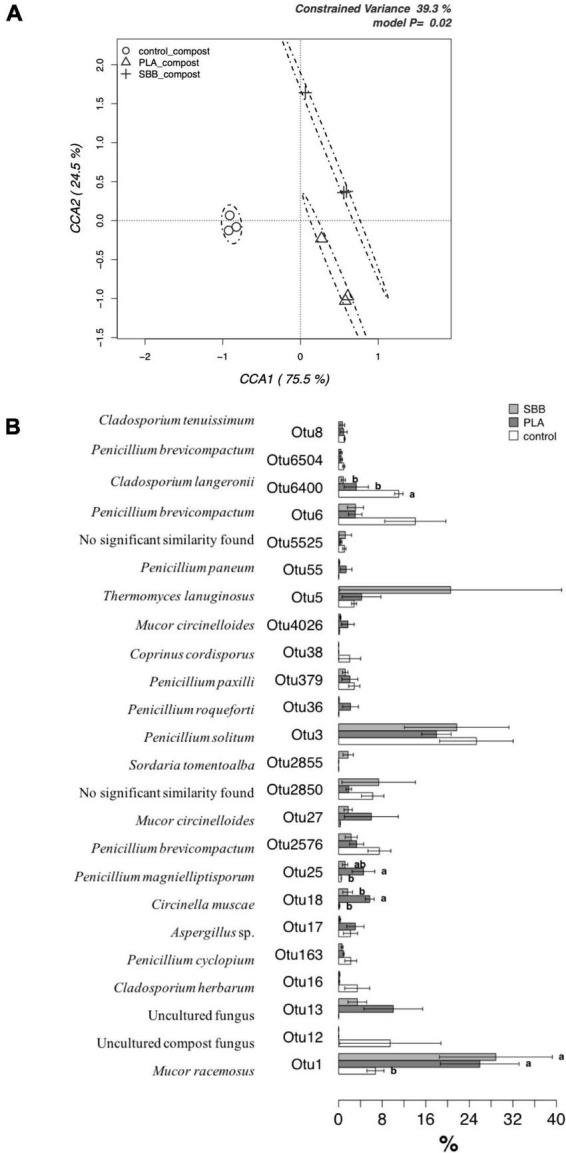
Fungal community in compost. **(A)** Hypothesis-driven canonical correspondence analysis (CCA) model performed on the total fungal OTUs relative abundance data from composts of each treatment (OFMSW, control; PLA; SBB). **(B)** Metastats model on the relative abundances of genera comprising 99.9% of each treatment’s fungal diversity in composts (OFMSW, control; PLA; SBB). OTUs showing significant differences according to the false discovery rate correction are highlighted with letters. PLA, polylactic acid; SBB, starch-based bioplastic; OFMSW, organic fraction of municipal solid waste.

To better evaluate the effects of bioplastics on the fungal community during aerobic composting, a db-RDA model based on OTUs was applied. Figure 8A shows the CCA that was significant (*p* = 0.02) and had a 39.3% of explanation of total variance. The first and the second canonical axes represented 75.5 and 24.5% of the variance, respectively. Composts obtained from the OFMSW showed a limited microbial diversity, and its ellipsis was located approximately in the center of the plot. Otherwise, the composts obtained from the treatment of the two bioplastics reported a wider fungal diversity, which was both positively correlated with the CCA1 axes.

In order to identify any significant differences between composts in terms of their distribution of the most abundant OTUs, a detailed analysis was carried out using a Metastats model (Figure 8B). *M. racemosus* (OTU1) was the only fungal OTU detected even in the anaerobic digestion and with a higher abundance than the others in several samples. In the compost from SBB and PLA, it was found with an abundance >24%, resulting statistically different from the positive control. Since different strains can produce lipase, gelatinase, xylanase, amylase, and caseinase enzyme (Silawat et al., 2013), highlighting its biodegradable role in composting process. Its greater abundance in compost from bioplastics treatment may be due to the higher carbon availability from the polymers used. The *Cladosporium* genus has the ability to produce cellulolytic and xylanolytic enzymes (Gupta et al., 2003; Fapohunda, 2006) and was also identified as PLA colonizing fungi (Karamanlioglu et al., 2014), but only the OTU6400 (*Cladosporium langeronii*) was found in abundance in PLA compost. The OTUs identified belonging to the genus *Penicillium*, being a good producer of various extracellular enzymes, is considered a crucial decomposer-recycler of organic matter of all types (Hee-Yeon et al., 2007; Li and Zong, 2010). Furthermore, *Penicillium solitum* (OTU3) was among the most abundant OTU, accounting for more than 16–24% of sequences for the three composts without statistically significant differences. This fungus was isolated from domestic compost and it is exploited for its extracellular lipases, which are more active on longer chained substrates (Chinaglia et al., 2014). However, there are no studies in the literature on the possible relationship of this species with bioplastics. On the contrary, some OTUs were particularly abundant in samples from bioplastics treatment, hypothesizing that they may play a key role in their biodegradation in thermophilic composting environments. On the one hand, OTU18 (*Circinella muscae*), OTU25 (*Penicillium magnielliptisporum*), and OTU27 (*Mucor circinelloides*) were mainly found in PLA compost, but no reference to the possible affinity of these fungi with bioplastics was found in the literature. On the other hand, *Penicillium roqueforti* (OTU36), due to its ability to totally assimilate DL-lactic acid, partially soluble racemic oligomers, and nitrogen source, was isolated as PLA-degrading microorganism (Torres et al., 1996). Since it was found only in the compost obtained from this treatment, its role may be to degrade PLA with promising potentials. In composts obtained from SBB treatment, OTU2855 (*Sordaria tomentoalba*) and OTU5 (*Thermomyces lanuginosus*) were more abundant compared to the other samples. *T. lanuginosus* is a polyester-degrading thermophilic bacterium able to hydrolyze the aromatic poly(trimethylene terephthalate) (PTT) (Eberl et al., 2008) and polyurethane (PU) (Zafar et al., 2014), but there are no references in the literature about this fungus associated with SBB. To summarize, our results suggested that PLA and SBB may influence and modulate the fungal community involved during the thermophilic aerobic composting of OFMSW.

## Conclusion

This work gain insight into the microbial structures during the anaerobic digestion and aerobic composting of OFMSW with PLA and SBB, giving a new approach to understanding the microbial activity of bacteria, archaea, and fungi involved and hypothesizing the relationship between these microorganisms and the different tested materials. Our results suggest that these materials shape microbial communities at different stages of the process, but their effect is most evident in the aerobic composting phase. Distinctive bacteria and fungi were detected in PLA and SBB treatment, suggesting a possible role of these materials in establishing different substrate conditions for their growth probably due to the presence and/or release of additives, chemicals, and plasticizers. Among fungi, *P. roqueforti* was found only in compost from PLA treatment and *T. lanuginosus* in that from SBB. Moreover, through a culturable isolation approach, the microorganisms found on each specific material may be exploited to enhance their biodegradation in contaminated environments. Certainly, a fundamental role is also played by the process parameters, which define the primary conditions for the growth of the different microbial groups involved. To better understand the fate and impacts of these biopolymers, further studies should focus on the microbial activity in the process and the feasibility of allowing these materials in food waste collection management. It is critical to understand whether bioplastics may lead to operational challenges not only in physical biodegradation but also in microbiological aspects, and the full extent of biodegradability of bioplastics must be discussed so that the existing plants can determine how to better process them.

## Data availability statement

The datasets presented in this study can be found in online repositories. The names of the repository/repositories and accession number(s) can be found below: NCBI – PRJNA883589.

## Author contributions

FB: conceptualization, methodology, formal analysis, and writing—original draft. FV: conceptualization and methodology. MS and SP: methodology—anaerobic digestion tests. CM, GB, and ET: methodology. PC: conceptualization, writing—review and editing, and supervision. EP: conceptualization, software, formal analysis, resources, writing—review and editing, supervision, project administration, and funding acquisition. All authors contributed to the article and approved the submitted version.

## Footnotes

1
http://hannonlab.cshl.edu/fastx_toolkit/

2
http://www.R-project.org/
