# Supplementary material for: Rigid bioplastics shape the microbial communities involved in the treatment of the organic fraction of municipal solid waste
Source: Front Microbiol. 2022 Nov 11;13:1035561. doi: 10.3389/fmicb.2022.1035561 (PMC9691671; doi:10.3389/fmicb.2022.1035561)
Supplement: Supplementary file 1 [file Data_Sheet_1.docx]

Supplementary Material

## Supplementary Figures

**Supplementary Figure 1.** Description of the whole experiment. Legend: PLA, polylactic acid; SBB, starch-based bioplastic; OFMSW, organic fraction of municipal solid waste.

## Supplementary Tables

**Table 1.** Average (± standard deviation) of α-diversity indexes and richness of bacterial microbiomes. The reported data are the average of the three replicates. ANOVA significant differences were indicated by F values (ns, *P<0.05, **P<0.01, ***P<0.005) for comparisons between rows. Data followed by the same minor letter on each row are not statistically different from each other (LSD test, P < 0.05). Legend: PLA, polylactic acid; SBB, starch-based bioplastic; OFMSW, organic fraction of municipal solid waste.

| Bacterial α-Diversity Indexes | | | | | | |
| --- | --- | --- | --- | --- | --- | --- |
|  | **Sample** | **Coverage** | **Chao** | **Sobs** | **Simpson** | **Shannon** |
|  | **Digestate*** | 0.993 | 446.980±52.18;d | 256±5.66;d | 0.024±0;abc | 2.891±0.19;abc |
|  | **OFMSW Slurry** | 0.984 | 959.398±76.47;a | 448.667±19.86;a | 0.02±0;abc | 3.093±0.04;a |
| Anaerobic digestion | **T0** | 0.99 | 554.184±39.13;cd | 301.333±4.16;bcd | 0.011±0;c | 2.458±0.04;bcde |
|  | **T1 OFMSW*** | 0.99 | 602.284±49.90;cd | 310.5±6.36;bcd | 0.013±0;abc | 2.616±0.05;abcde |
|  | **T1 PLA** | 0.989 | 657.795±62.94;c | 316±5.57;bc | 0.013±0;bc | 2.633±0.03;abcd |
|  | **T1 SBB** | 0.991 | 592.317±50.25;cd | 290.333±11.5;bcd | 0.013±0;abc | 2.546±0.03;bcde |
|  | **T2 OFMSW** | 0.99 | 620.918±131.66;cd | 295.333±6.81;bcd | 0.012±0;c | 2.395±0.03;cde |
|  | **T2 PLA** | 0.99 | 586.179±78.86;cd | 281.667±13.05;cd | 0.013±0;bc | 2.437±0.07;bcde |
|  | **T2 SBB** | 0.991 | 593.246±34.89;cd | 286.667±4.73;bcd | 0.014±0;abc | 2.504±0.03;bcde |
|  | **Tfinal OFMSW** | 0.989 | 638.989±41.1;c | 334±13.11;b | 0.014±0;abc | 2.596±0.01;bcde |
|  | **Tfinal PLA** | 0.99 | 616.292±45.98;cd | 296.667±6.51;bcd | 0.013±0;abc | 2.307±0.06;cde |
|  | **Tfinal SBB** | 0.989 | 677.513±85.36;c | 311±36.51;bc | 0.01±0;c | 2.138±0.18;e |
| Composting | **Compost OFMSW** | 0.989 | 688.529±183.21;bc | 293.333±82.95;bcd | 0.028±0.01;a | 2.859±0.2;ab |
|  | **Compost PLA** | 0.988 | 848.731±133.75;ab | 275±48.66;cd | 0.019±0.01;abc | 2.214±0.35;de |
|  | **Compost SBB** | 0.989 | 708.049±157.38;bc | 294.333±3.79;bcd | 0.026±0.03;ab | 2.455±0.9;bcde |
| *F-value* |  |  | *4.450**** | *6.590**** | *1.282, ns* | *2.413** |

**Table 2.** Average (± standard deviation) of α-diversity indexes and richness of archaeal microbiomes. The reported data are the average of the three replicates. ANOVA significant differences were indicated by F values (ns, *P<0.05, **P<0.01, ***P<0.005) for comparisons between rows. Data followed by the same minor letter on each row are not statistically different from each other (LSD test, P < 0.05). Legend: PLA, polylactic acid; SBB, starch-based bioplastic; OFMSW, organic fraction of municipal solid waste.

| Archaeal α-Diversity Indexes | | | | | | |
| --- | --- | --- | --- | --- | --- | --- |
|  | **Sample** | **Coverage** | **Chao** | **Sobs** | **Simpson** | **Shannon** |
|  | **Digestate** | 0.991 | 5±1.73;b | 3.333±0.58;e | 0.311±0.05;a | 0.096±0.02;g |
| Anaerobic digestion | **T0** | 0.982 | 17.833±17.47;ab | 7±2.65;de | 0.171±0.05;bcd | 0.334±0.09;ef |
|  | **T1 OFMSW** | 0.983 | 15.833±12.33;ab | 8±1.73;bcd | 0.151±0.04;cd | 0.449±0.1;de |
|  | **T1 PLA*** | 0.977 | 16.267±1.32;ab | 12.5±0.71;a | 0.106±0.01;d | 0.741±0.05;bc |
|  | **T1 SBB*** | 0.971 | 23.375±10.78;ab | 12.500±0.71;a | 0.099±0.01;d | 0.645±0.02;cd |
|  | **T2 OFMSW** | 0.985 | 14.333±4.16;ab | 8±1;bcd | 0.167±0.01;bcd | 0.616±0.11;cd |
|  | **T2 PLA*** | 0.992 | 6.5±0.71;ab | 5.500±0.71;de | 0.244±0.01;abc | 0.537±0.24;cde |
|  | **T2 SBB** | 0.99 | 8.583±3.17;ab | 7.333±2.08;cd | 0.18±0.05;bcd | 0.517±0.11;cde |
|  | **Tfinal OFMSW** | 0.974 | 28.5±20.46;a | 12.333±3.06;a | 0.126±0.03;d | 0.887±0.17;b |
|  | **Tfinal PLA** | 0.977 | 24.833±20.06;ab | 11.333±0.58;ab | 0.118±0.02;d | 0.724±0.14;bc |
|  | **Tfinal SBB** | 0.982 | 17.333±11.06;ab | 11±4;abc | 0.193±0.08;bcd | 1.127±0.14;a |
| Composting | **Compost OFMSW** | 0.982 | 11.5±4.27;ab | 6.333±2.08;de | 0.18±0.07;bcd | 0.225±0.09;fg |
|  | **Compost PLA** | 0.99 | 10±11.27;ab | 5±2.65;de | 0.25±0.11;ab | 0.22±0.11;fg |
|  | **Compost SBB** | 0.979 | 21.333±16.65;ab | 7.667±3.79;bcd | 0.165±0.06;bcd | 0.359±0.2;ef |
| *F-value* |  |  | *0.903, ns* | *4.434*** | *3.248*** | *14.299**** |

**Table 3.** Average (± standard deviation) of α-diversity indexes and richness of fungal microbiomes. The reported data are the average of the three replicates. ANOVA significant differences were indicated by F values (ns, *P<0.05, **P<0.01, ***P<0.005) for comparisons between rows. Data followed by the same minor letter on each row are not statistically different from each other (LSD test, P < 0.05). Legend: PLA, polylactic acid; SBB, starch-based bioplastic; OFMSW, organic fraction of municipal solid waste.

| **Fungal α-Diversity Indexes** | | | | | | |
| --- | --- | --- | --- | --- | --- | --- |
|  | **Sample** | **Coverage** | **Chao** | **Sobs** | **Simpson** | **Shannon** |
|  | **Digestate** | 0.994 | 400.777±54.17;de | 268±15.87;bcde | 0.078±0.04;cd | 4.074±0.21;ab |
|  | **OFMSW Slurry** | 0.988 | 663.565±19.48;ab | 513.333±31.56;a | 0.038±0;fg | 4.17±0.03;ab |
| **Anaerobic digestion** | **T0 *** | 0.993 | 459.708±26.93;cde | 240.5±9.19;bcde | 0.117±0.02;a | 4.145±0.01;ab |
|  | **T1 OFMSW** | 0.995 | 387.662±77.31;de | 307±80.61;bcd | 0.105±0.03;ab | 4.361±0.38;a |
|  | **T1 PLA** | 0.993 | 489.664±94.94;cd | 242±38.63;bcd | 0.096±0.01;bc | 4.097±0.12;ab |
|  | **T1 SBB** | 0.991 | 559.191±122.81;bc | 283±19.08;b | 0.076±0.01;cd | 4.115±0.07;ab |
|  | **T2 OFMSW** | 0.994 | 370.063±10.23;de | 278.333±47.23;cde | 0.066±0.03;ef | 3.894±0.41;de |
|  | **T2 PLA** | 0.994 | 393.618±34.63;de | 219±11.27;cde | 0.049±0.01;fg | 3.459±0.16;e |
|  | **T2 SBB*** | 0.995 | 322.781±45.65;e | 211.5±13.44;de | 0.04±0;fg | 3.270±0.11;e |
|  | **Tfinal OFMSW** | 0.993 | 470.147±150.13;cd | 256.6±31.83;bcd | 0.058±0.02;cd | 3.669±0.51;abc |
|  | **Tfinal PLA** | 0.995 | 408.238±82.26;de | 272.333±45.45;bcd | 0.073±0.01;cde | 4.045±0.3;ab |
|  | **Tfinal SBB** | 0.993 | 490.398±53.03;cd | 281.333±6.66;bc | 0.059±0.01;def | 3.896±0.09;bc |
| **Composting** | **Compost OFMSW** | 0.983 | 783.907±65.01;a | 555±50.51;a | 0.022±0;g | 3.721±0.21;cd |
|  | **Compost PLA** | 0.992 | 371.989±42.23;de | 235.667±8.62;cde | 0.035±0.01;fg | 2.835±0.09;f |
|  | **Compost SBB** | 0.994 | 318.613±83.73;e | 208.667±48;e | 0.022±0.01;g | 2.401±0.05;g |
| *F-value* |  |  | *8.206**** | *24.107**** | *9.745**** | *26.968**** |
